# Antibacterial, Antioxidant, Larvicidal and Anticancer Activities of Silver Nanoparticles Synthesized Using Extracts from Fruits of *Lagerstroemia speciose* and Flowers of *Couroupita guianensis*

**DOI:** 10.3390/molecules27227792

**Published:** 2022-11-12

**Authors:** Venkatadri Babu, Selvaraj Arokiyaraj, Swathi Pon Sakthi Sri, Mary George, Rameshkumar Marimuthu Ragavan, Dinesh Dharmalingam, Taehwan Oh, Subramaniyan Ramasundaram, Paul Agastian

**Affiliations:** 1 Department of Plant Biology and Biotechnology, Loyola College (Autonomous), Affiliated to University of Madras, Chennai 600034, Tamil Nadu, India; 2 Department of Food Science & Biotechnology, Sejong University, Seoul 05006, Korea; 3 Department of Chemistry, Stella Maris College (Autonomous), Affiliated to University of Madras, Chennai 600017, Tamil Nadu, India; 4 Department of Microbiology and Biotechnology, Presidency College (Autonomous), Affiliated to University of Madras, Chennai 600005, Tamil Nadu, India; 5 Division of Vector Control, Entomology Research Institute, Loyola College (Autonomous), Affiliated to University of Madras, Chennai 600034, Tamil Nadu, India; 6 School of Chemical Engineering, Yeungnam University, Gyeongsan 38541, Korea

**Keywords:** *Lagerstroemia speciose*, *Couroupita guianensis*, silver nanoparticles, antibacterial, larvicidal, anticancer, green synthesis

## Abstract

The present study aimed to analyze the in vitro antibacterial, antioxidant, larvicidal and cytotoxicity properties of green synthesized silver nanoparticles (Ag NPs) using aqueous extracts from fruits of Lagerstroemia speciosa and flowers of *Couropita guinensis*. Synthesized Ag NPs were characterized using UV-DRS, FTIR, XRD, DLS, and High-Resolution SEM and TEM analyses. Absorption wavelength was observed at 386 nm by UV-DRS analysis and energy band gap was calculated as 3.24 eV. FTIR analysis showed the existence of various functional groups in the aqueous extract and in the NPs. DLS analysis showed the stability and particle size of the synthesized Ag NPs. SEM analysis revealed that Ag NPs are in a face centered cubic symmetry and spherical shape with a size of 23.9 nm. TEM analysis showed particle size as 29.90 nm. Ag NPs showed antibacterial activity against both Gram-positive and Gram-negative bacteria. DPPH scavenging trait of Ag NPs was ranging from 20.0 ± 0.2% to 62.4 ± 0.3% and observed significant larvicidal activity (LC50 at 0.742 ppm and LC90 at 6.061 ppm) against Culex quinquefasciatus. In vitro cytotoxicity activity of Ag NPs was also tested against human breast cancer (MCF-7) and fibroblast cells (L-929) and found that cells viabilities are ranging (500 to 25 µg/mL) from 52.5 ± 0.4 to 94.0 ± 0.7% and 53.6 ± 0.5 to 90.1 ± 0.8%, respectively. The synthesized Ag NPs have the potential to be used in the various biomedical applications.

## 1. Introduction

Green synthesis of nanoparticles (NPs) is a relatively safe, more cost-effective and eco-friendly method than physical and chemical methods [1]. The NPs of metals such as Au, Ag, Zn, and Cu have been synthesized and explored for various medicinal applications. Among these metal NPs, Ag NPs have been widely synthesized using plant-based extracts. Ag NPs are known for their unique properties including superior thermal and electrical conductivities, greater stability and promising bioactivities [2]. Bio-synthesized Ag NPs are receiving considerable attention in the biomedical fields, especially drug development against infectious diseases, drug delivery, diagnostics, and mosquito control [3]. Ag NPs with a size range of 1–100 nm along with high surface area and a larger extent of surface reactive canters attain great attention [4,5,6,7,8].

In this study, fruits of *Lagerstroemia speciosa (L. specieosa)* and flowers of *Couroupita guianensis (C. guianensis)* were used for the synthesis of Ag NPs. The plant *L. speciosa*, commonly known as “Jarul”, belongs to the Lythraceae family [9]. These plant parts are containing various phytocomponents such as alkaloids, terpenoids, flavonoids and others [10,11]. Further, its leaf and flower extract has been widely studied for green synthesis of Ag NPs [8] and studied for various biological activities, such as antibacterial, anti-diabetic and anti-inflammatory activities [12,13]. Besides that, parts of *C. guianensis* (common name is ayahuma and cannonball tree), the plant belonging to the Lecythidaceae family, were also explored for anticancer, antifungal and anti-inflammatory applications. Extracts of this plant parts have been used for treating common cold, stomach ache and malaria [14,15]. Recently, *C. guianensis* leaf and fruit extracts were reported as useful for rapid and cost-effective synthesis of Ag NPs and to control the dengue vector *Aedes aegypti* [16]. However, the efforts taken to study the biological activities of Ag NPs synthesized from plant extracts were limited. Therefore, in the present study, an attempt has been made to investigate the in vitro antibacterial, antioxidant, larvicidal and cytotoxicity properties of Ag NPs using extracts from fruits of L. specieosa and flowers of C. guianensis. The combination of functional phytochemicals from this mixture can yield Ag NPs with wide range of biological activities.

## 2. Results and Discussion

**Figure 1:**
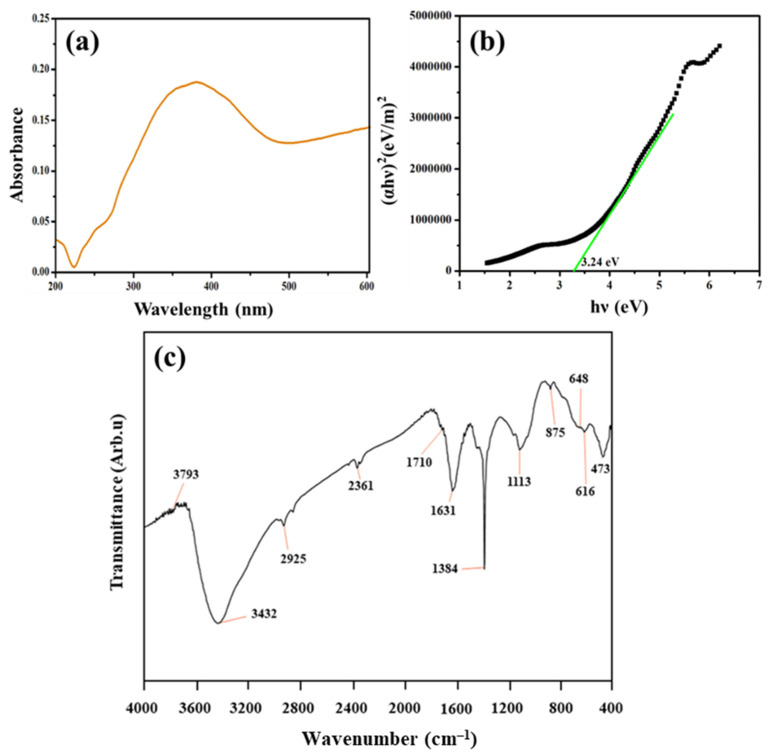
Chemical structure and optical band gap of green synthesized Ag NPs: (**a**) UV-Vis spectrum; (**b**) Optical band gap estimated by Tauc plot, and (**c**) FTIR spectrum.

Ultraviolet-Visible Diffused Reflectance Spectroscopy (UV-DRS) is most extensively used techniques for structural characterization of metal NPs. As shown in Figure 1a, the surface plasmon resonance (SPR) of Ag NPs emerged at around 400 nm. This peak confirmed the reduction of silver nitrate into Ag NPs. Ag NPs have an SPR peak in this area, which could be due to spherical nanoparticles [17,18]. Further, the direct optical band gap obtained using the Tauc plot is shown in Figure 1b. The direct optical bandgap energy of the synthesized Ag NPs was found to be 3.24 eV.

Fourier transform Infrared (FTIR) spectroscopy is a powerful tool for identifying the functional groups involved in nanoparticle bio-reduction and stability. FTIR spectrum (Figure 1c) showed major infrared transmittance peaks at 3431, 2925, 1631, and 1384 cm^−1^ corresponding to O–H, C–H, C–N/C–C, and N=O, respectively. The OH peak was aroused from alcohols and phenolic compounds with strong hydrogen bonding. The phenolic compounds were the main contributors to the bio-reduction process [19,20,21,22]. The presence of C–N and C–C stretching vibrations of amide linkage and protein molecules involved in the synthesis and encapsulation of Ag NPs was confirmed by a strong peak found at 1631 and 1384 cm^−1^ [23,24,25,26,27]. The FTIR spectrum of biosynthesized Ag NPs coincides with the Ag NPs synthesized using *Urtica dioica* Linn. leaves, where Ag NPs were found to be coated with the residues of phenolic compounds, amino acids and peptides [28]. These vibrational transmittance peaks revealed that the Ag NPs were capped by the secondary metabolites from plant sources. Table 1 shows the functional group assignments and their force constant values.

**Figure 2:**
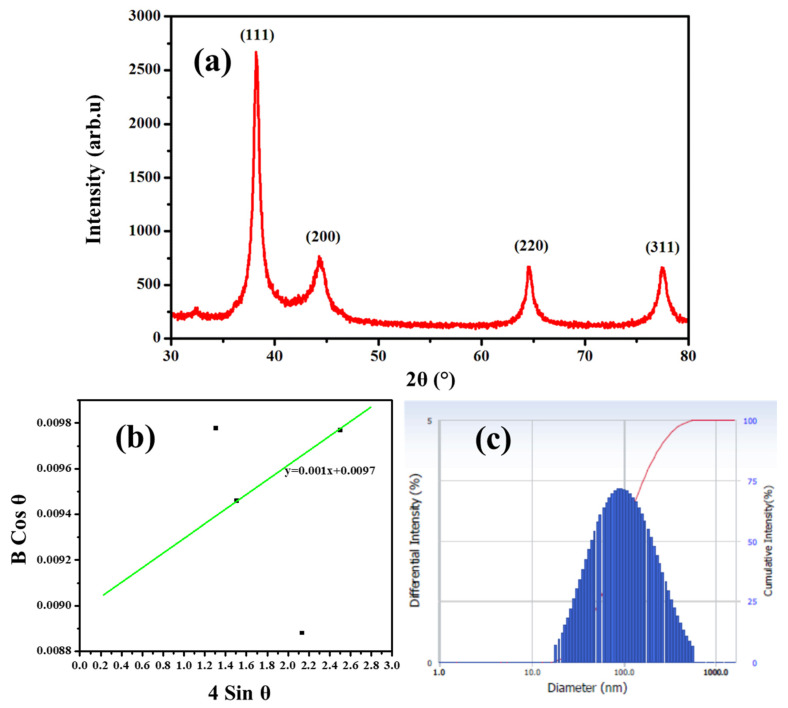
Crystalline structure, grain size, and particle size of green synthesized Ag NPs: (**a**) XRD pattern; (**b**) W-H plot; and (**c**) DLS plot.

X-ray diffraction pattern (XRD) is used to determine the crystallite size and crystalline nature of NPs. Figure 2a shows the XRD pattern of green synthesized Ag NPs. Four strong characteristic peaks were observed at 2θ = 38.23°, 44.20°, 64.60°, and 77.54°, which are corresponding to (111), (200), (220), and (311) diffraction planes of Ag, respectively. It can be indexed to the facets of the face-centered cubic crystal structure of Ag [29]. The XRD pattern exhibited greater preferential orientation at 2θ = 38.23° corresponding to (111) reflection plane of Ag NPs. The crystallographic planes obtained were consistent with the joint committee on powder diffraction standards (JCPDS card no. 89–3799) [30,31]. No other diffraction peaks associated with other elements were observed in the XRD pattern, which indicated that the precursor material silver nitrate has been completely converted to Ag NPs and the phase purity of the synthesized Ag NPs. A tiny peak at 32.5° could be attributed to AgO. Further, the Williamson–Hall (W–H) method plot (Figure 2b) was drawn with 4Sinθ along the x-axis and βCosθ along the y-axis to calculate crystallite size and strain. The crystallite size and microstrain estimated according to the W–H method were 14.29 nm and 10 × 10^−4^, respectively. Table 2 lists the physical parameters acquired from XRD analysis. Dynamic light scattering (DLS) studies were used to analyze the particle size in the colloidal solution by irradiating light sources. The temperature in the measurement chamber was kept at 25 °C. The average size of the particles was estimated as 76.4 nm by the DLS histogram (Figure 2c). Its polydispersity index was 0.325 and its diffusion coefficient was 6.438 × 10^8^ cm^2^/s. The green synthesized Ag NPs in this study were found to be polydispersed, according to the size distribution curve.

**Figure 3:**
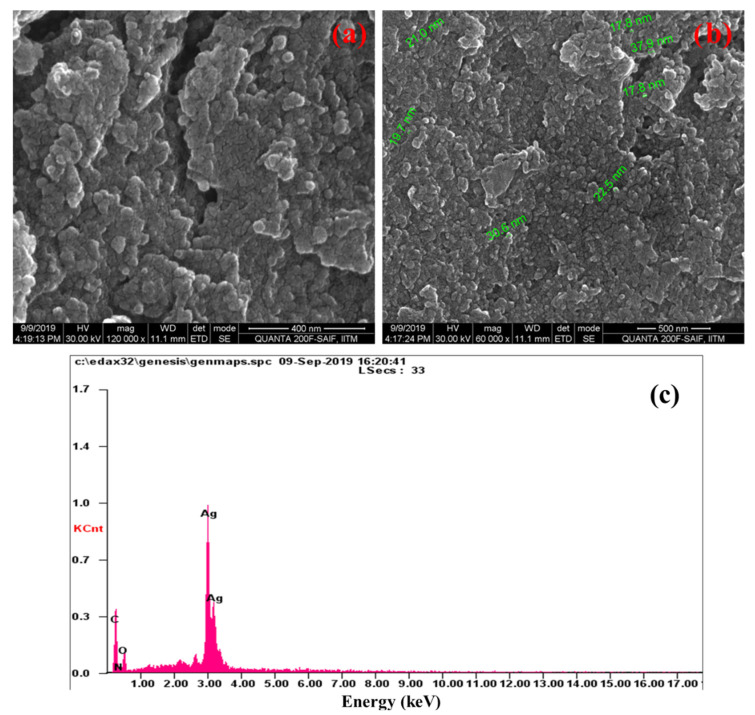
Morphology and elemental composition of green synthesized Ag NPs: (**a**,**b**) HRSEM images, and (**c**) EDX spectrum.

The morphology and size of the Ag NPs were characterized using High Resolution Scanning Electron Microscope (HR-SEM). It was found that the synthesized Ag NPs were spherical in shape and the size ranging from 17 to 37 nm. The average particle size of Ag NPs was 23.9 nm. The particles were evenly distributed in a uniform pattern (Figure 3a,b). Furthermore, the Energy dispersive X-ray (EDX) spectroscopy is a useful tool for assessing the elemental composition. A strong signal for metallic silver was observed at 2.98 keV [32]. In addition to major peak of Ag, the weak signals attributable to the presence of C, N and O (Figure 3c) were also seen. These peaks were corroborated with the presence of phytochemicals capped on Ag NPs.

**Figure 4:**
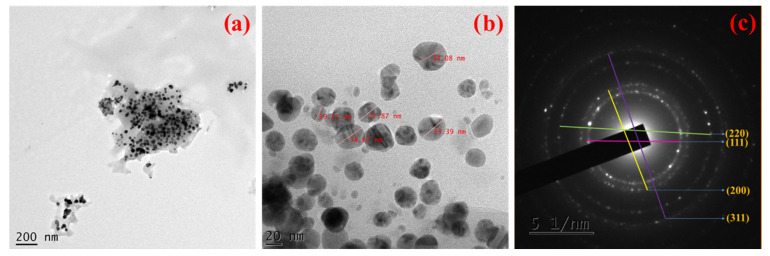
Morphology and crystalline pattern of green synthesized Ag NPs: (**a**,**b**) HRTEM images, and (**c**) SAED pattern.

The green synthesized Ag NPs also appeared in spherical in the images (Figure 4a,b) obtained from High resolution Transmission electron microscope (HR-TEM). In HR-TEM images with various magnifications, the Ag NPs were uniformly dispersed and their diameter was about 29.9 nm. The HR-TEM results coincided with the finding obtained from HR-SEM studies. In addition, Selected Area (Electron) Diffraction (SAED) pattern is useful to obtain further insight on the crystalline nature and lattice parameters. In SAED pattern (Figure 4c), Ag NPs appeared polycrystalline in nature. The bright concentric rings with little dots were also seen. These characteristic diffraction rings were indexed as (111), (200), (220), and (311) planes which were identical to the face-centered cubic lattice structure typically observed for Ag NPs. Notably, the observations from SAED pattern were consistent with the XRD results.

**Figure 5:**
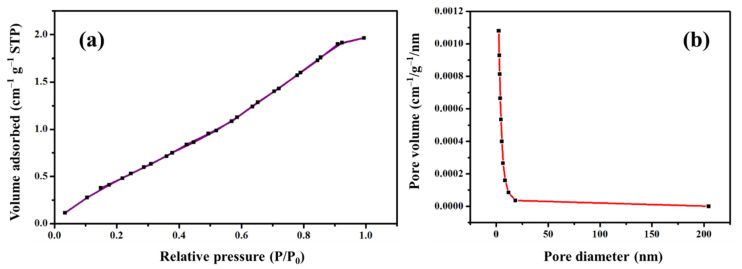
Surface area of pore size of green synthesized Ag NPs: (**a**) Nitrogen adsorption–desorption isotherm, and (**b**) Barret–Joyner–Halenda (BJH) pore size distribution plot.

A Brunner–Emmett–Teller (BET) surface area analysis was used to assess the surface area, pore size, and pore volume Ag NPs. The green synthesized Ag NPs have a surface area of 4.208 m^2^/g. With monolayer adsorption, the nitrogen adsorption–desorption isotherm for Ag NPs (Figure 5a) was Type I pseudo-Langmuir. The characterization of microporous materials, those with pore diameter of less than 2 nm, follows the Type I isotherm model. The Barre–Joyner–Halenda (BJH) pore size distribution plot depicted in Figure 5b exhibited a pore volume of 0.004 cm^3^/g and pore diameter (d) = 1.980 nm. The pore diameter of less than 2 nm is distinctive of microporous materials. The pores were supramicropores in nature as their (d) value was ranged between 0.7 and 2 nm. 

The Ag NPs synthesized using the combination of extracts from the fruits of *L. specieosa*, and flowers of *C. guianensis* showed a broad spectrum of antibacterial activity against both Gram (+) and Gram (−) bacteria. Ag NPs showed potential bactericidal activity against Enterococcus faecalis (15.3 ± 0.5), Proteus mirabilis (14.3 ± 0.5), Staphylococcus aureus (11.6 ± 0.5) and Yersinia enterocolitica (12.3 ± 1.1) at the concentrations ranging from 0.5 to 2.5 µg/mL (Table 3). Demirbas et al. reported that the green synthesized Ag NPs showed more effective antimicrobial activity than that of the plant extracts used [33].

**Figure 6:**
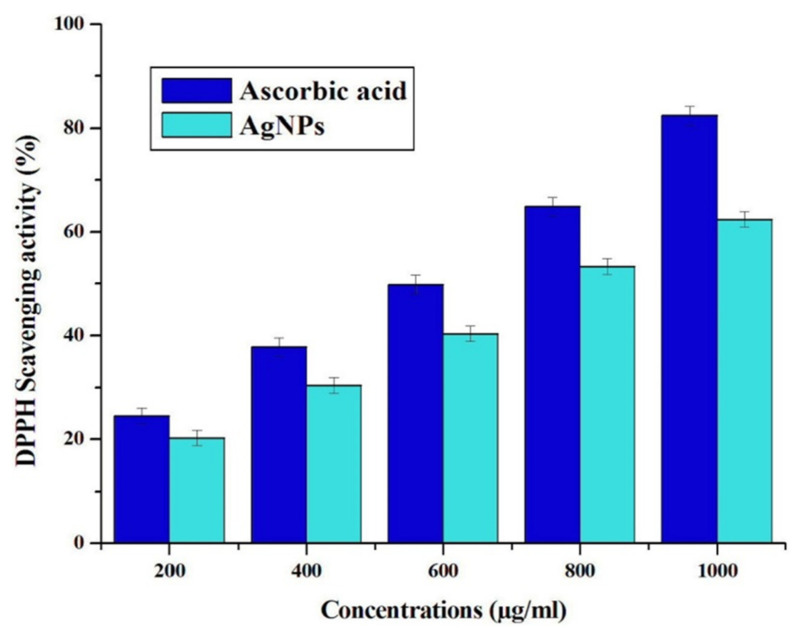
DPPH radical scavenging activity of Ag NPs assessed using ascorbic acid.

In the in vitro antioxidant activity, the DPPH degrading properties of Ag NPs synthesized from synergistic extracts of fruits of *L. specieosa* and flowers of *C. guianensis* were shown in Figure 6. The DPPH degrading properties of synthesized Ag NPs were found to increase with the increasing concentration of NPs (200 to 1000 μg/mL) (Figure 6). DPPH scavenging trait of Ag NPs was ranging from 23.9 ± 0.5% to 81.6 ± 1.0% and ascorbic acid was ranging from 20.0 ± 0.2% to 62.4 ± 0.3%. The DPPH degrading properties provide an easy and rapid method for estimating the free radical scavenging activity of green synthesized Ag NPs. The colour changes from purple to yellow after reduction by Ag NPs, which can also be confirmed by the decrease in absorbance at 517 nm [34].

The synthesized Ag NPs in this study were examined for larvicidal activity. It was found that Ag NPs showed significant larvicidal activity and LC50 and LC90 values were noted at the concentration of 0.742 and 6.061 ppm against *Culex quinquefasciatus*, respectively (Table 4). Ag NPs showed a maximum of 70% larvicidal activity against the tested larvae of *Culex quinquefasciatus* at 2 ppm concentration. The treated larvae exhibited restless movement and convulsion followed by death. There was no larval mortality in the controls, and all of the larvae were active and moved normally. In a study by Cecilia et al. [35], ecbolin A and ecbolin B compounds isolated from *Ecbolium viride* were tested against third instar larvae of *Culex quinquefasciatus* with the LC50 at the concentration of 7.22 and 1.36 ppm and LC90 at 14.49 and 2.76 ppm, respectively.

**Figure 7:**
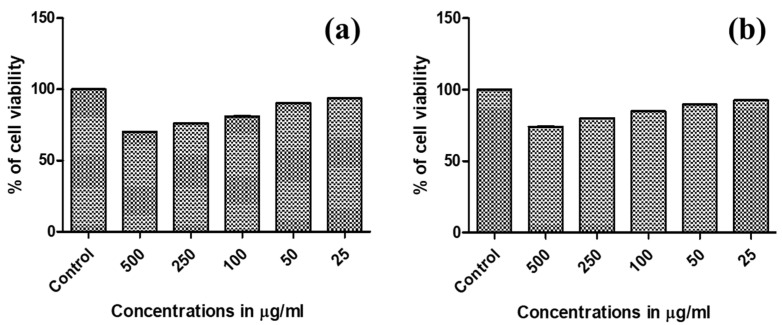
In vitro cytotoxicity effect of green synthesized Ag NPs: (**a**) Cytotoxicity against MCF-7 cells; and (**b**) Cytotoxicity against L-929 (**b**) cells.

In the present study, synthesized Ag NPs were evaluated for their in vitro cytotoxicity property against MCF-7, L929, and the normal (fibroblast) cell lines. Ag NPs depicted strong anticancer activity against both the MCF-7 and L929 cell lines with cells viabilities ranging from 92.5 ± 0.4 to 74.0 ± 0.7% and 93.6 ± 0.5 to 70.1 ± 0.8%, respectively (Figure 7a,b). Cytotoxicity studies performed with Ag NPs synthesized using the extract of *Melia azedarach* Linn leaves confirmed that the cell viability was decreased due to apoptosis induced cell death [36]. The extract of *L. specieosa fruits* and *C. guianensis* flowers known to have antibacterial and anti-viral activities. Ag NPs coated with ingredients in this extract, possibly favored the apoptosis induced cell death, thereby, decrease in cell viability was witnessed.

## 3. Materials and Methods

### 3.1. Synthesis of Ag NPs

Silver nitrate (Ag NO_3_) was purchased from Merck Company. Fruits of *L. specieosa* and flowers of *C. guianensis* were collected from Loyola College, Chennai. Five grams of mixed plant powder were added in 100 mL of milli Q water and boiled for 5 min. Solid debris in the boiled extract was removed using Whatman No.1 filter paper and centrifugation (3000 rpm for 10 min). The extract solution was used for the synthesis of Ag NPs. Then, 1M Ag NO_3_ solution (20 mL) was added to 80 mL of filtered plant extract. Upon stirring, an immediate colour change of pale brown to dark intense brown was observed. The precipitate was washed using milli Q water by centrifugation and dried in a vacuum desiccator.

### 3.2. Physiochemical Characterization of Ag NPs

Prior to physiochemical characterization, the green synthesized Ag NPs sample was dried at 60 °C under vacuum. Ultraviolet-visible spectrum of Ag NPs was recorded using Jasco V-750 (India) UV-Vis spectrophotometer. FTIR spectrum was obtained using Perkin Elmer Spectrum Two (USA) FTIR spectrophotometer. The scanning range and resolution were 4000–400 cm^−1^ and 4 cm^−1^, respectively. The crystalline structure of the Ag NPs was investigated by X-ray diffraction studies (D8 Advance Bruker X-ray diffractometer, USA). The X-ray diffractometer was operated at the voltage of 40 kV and current of 40 mA. The radiation source was Cu/kα (λ = 1.5412 Å). The size distribution of Ag NPs was evaluated using DLS (Micromeritics Nano Plus, Germany). HR-SEM images were obtained using FEI Quanta FEG 200 high resolution scanning electron microscope equipped with the energy dispersive X-ray spectrophotometer. HR-TEM analysis was performed using Jeol/JEM 2100 high resolution transmission electron microscope operated at the accelerating voltage of 200 kV. For HR-TEM analysis, the samples were prepared by drop casting 10 µL of Ag NPs–ethanol dispersion onto a carbon coated copper grid. The surface area of the Ag NPs was determined by Brunauer–Emmett–Teller surface area analyzer (BET, Nova e2200 Quantachrome, India).

### 3.3. In vitro Antibacterial Assessment

Green synthesized Ag NPs were studied for their antibacterial potential against *Staphylococcus aureus* (MTCC-3615), *Enterococcus faecalis* (MTCC-439), *Yersinia enterocolitica* (MTCC-840), and *Proteus vulgaris* (MTCC-1771). Isolated bacterial colonies were inoculated in Muller–Hinton broth (MHB) and incubated at 37 °C for 24 h. Turbidity of broth culture was adjusted to 0.5 McFarland’s standards. Bacterial lawn culture was made on MH Agar medium using a cotton swab. The Kirby–Bauer disc diffusion method was used for screening the antibacterial activity of Ag NPs. Various concentrations (0.5–2 µg/mL) of Ag NPs were loaded on sterile paper disc followed by being placed on the MH agar surface containing lawn culture of bacteria and incubated at 37 °C for 24 h [37]. Streptomycin was used as a standard antibiotic control. The zone of inhibition around the disc was measured. The experiment was carried out in triplicates.

### 3.4. Antioxidant Activity

*DPPH Scavenging Assay.* The free radical scavenging activities of Ag NPs at varying concentrations (200–1000 μg/mL) were measured using a DPPH method [38]. Percentage (%) of DPPH scavenging properties of Ag NPs was calculated using the formula: DPPH scavenging capacity (%) = [(A_sample_−A_blank_)/A_control_] × 100.

### 3.5. Larvicidal Activity 

The third instar *Culex quinquefasciatus* larvae was used for the experiment. The larvicidal activity of synthesized Ag NPs was examined using the method designated by WHO (2005). The various concentrations of Ag NPs such as 0.5, 1.5 and 2 ppm were used for analysing larvicidal activity. A hundred early third instar larvae were introduced into the containers with different concentrations of Ag NPs. Water alone and DMSO in water were used as negative controls. Dead larvae were observed after 24 h. The percentage of mortality was calculated using the following formula: Number of dead larvae/Number of larvae introduced X 100. Corrections for mortality were done using the formula used by Murugan et al. [39,40], 1- n in T after treatment ×100/n in C after treatment ×100 when control mortality was below 5% (b). LC50 and LC90 values were estimated using US EPA probit analysis software (version 1.5).

### 3.6. Anti-Cancer Activity

The anti-cancer activity of synthesized silver nanoparticles against breast cancer cells (MCF-7) and fibroblast cells (L-929) were evaluated using MTT [3-(4,5-dimethylthiazol-2-yl)-2,5-diphenyltetrazolium bromide] assay [41,42]. The optical density (OD) was read and the cell viability (%) was calculated using the following formula: % of cell viability= [OD value of experimental sample/OD value of experimental control] × 100. 

### 3.7. Statistical Analysis

The results obtained from the study were analyzed and data were analyzed as means ± standard deviation. The *p* value < 0.05 was considered statistically significant.

## 4. Conclusions

In this focused study, Ag NPs were synthesized using synergistic extracts of fruits of *Lagerstroemia speciosa* and flowers of *Couroupita guianensis*. Instrumental analyses revealed that synthesized Ag NPs have face centered cubic symmetry with spherical morphology. The particle size was measured as 23.9 and 29.9 nm using HR-SEM and HR-TEM analysis, respectively. Ag NPs were polydispersed and polycrystalline in nature. FTIR results revealed the capping of phytochemicals on the surface of Ag NPs. The green synthesized Ag NPs exhibited significant antibacterial activity against both Gram-positive and Gram-negative bacteria, and also antioxidant activity. In addition, Ag NPs showed antioxidant activity. Furthermore, effective larvicidal activity against *Culex quinquefasciatus* and in vitro cytotoxic against MCF-7 cells was found. This study concludes that green synthesized Ag NPs has the potential to be used for therapeutic application and microbial disinfection. 

## Figures and Tables

**Table 1:** Functional groups present in the FTIR spectrum biosynthesized Ag NPs.

Peak (cm^−1^)	Assignment
3431	O–H
2925	C–H
1631	N–H
1384	C-N/C–H

**Table 2:** Grain size, dislocation density and strain values obtained from XRD analysis.

Grain Size from Scherrer Equation (nm)	Dislocation Density δ	Grain Size from W-H Plot (nm)	Strain ε (×10^−4^)
14.66	0.004653	14.29	10

**Table 3:** Lethal concentrations of green synthesized Ag NPs *against* Gram-positive and Gram-negative bacteria.

Organisms	Streptomycin (10 μg/disc)	Antibacterial Activity (mm)				
		0.5 (µg/mL)	1 (µg/mL)	1.5 (µg/mL)	2 (µg/mL)	2.5 (µg/mL)
*Staphylococcus aureus*	19.3 ± 1.5	8.6 ± 0.5	9.6 ± 0.5	10.3 ± 1.5	10.6 ± 0.5	11.6 ± 0.5
*Enterococcus faecalis*	18.6 ± 0.5	10.3 ± 1.1	11.3 ± 1.5	12.3 ± 1.5	14.6 ± 1.5	15.3 ± 0.5
*Yersinia enterocolitica*	15.3 ± 1.1	7.6 ± 0.5	7.3 ± 1.5	8.6 ± 0.5	9.6 ± 1.1	12.3 ± 1.1
*Proteus mirabilis*	19.6 ± 1.1	11.3 ± 1.1	11.6 ± 0.5	12.3 ± 2.0	13.6 ± 1.1	14.3 ± 0.5

**Table 4:** Lethal concentrations of green synthesized Ag NPs against the larvae of *Culex quinquefasciatus.*

Mosquito	Extracts	LC50 (ppm)	95% Confidence Limit		LC_90_ (ppm)	95% Confidence Limit		Intercept ± SE	Slope ± SE	χ^2^
			LL	UL		LL	UL			
*Cx. quinquefasciatus*	Ag NPs	0.742	0.392	0.834	6.061	4.582	26.124	3.7 ± 0.9	1.6 ± 0.4	3.9 *
	Temephos	1.74	1.56	1.83	5.02	5.42	4.93	2.5 ± 0.3	5.5 ± 0.1	4.7 *

* LC50—lethal concentration that kills 50% of the exposed larvae, LC90—lethal concentration that kills 90% of the exposed larvae, LL lower limit (95% confidence limit), UL upper limit (95% confidence limit). * *p* ≤ 0.05, level of significance of chi-square values.

## Data Availability

Upon reasonable request, the data supporting this investigation are available from the corresponding authors.

## Sample Availability

Upon reasonable request, Ag NPs synthesized in this work are available from the corresponding authors.
